# AI-Based Angle Map Analysis of Facial Asymmetry in Peripheral Facial Palsy

**DOI:** 10.3390/bioengineering13040426

**Published:** 2026-04-06

**Authors:** Andreas Heinrich, Gerd Fabian Volk, Christian Dobel, Orlando Guntinas-Lichius

**Affiliations:** 1 Department of Radiology, Jena University Hospital—Friedrich Schiller University, Am Klinikum 1, 07747 Jena, Germany; 2 Department of Otorhinolaryngology, Jena University Hospital—Friedrich Schiller University, Am Klinikum 1, 07747 Jena, Germany; fabian.volk@med.uni-jena.de (G.F.V.); christian.dobel@med.uni-jena.de (C.D.); orlando.guntinas@med.uni-jena.de (O.G.-L.); 3 Facial-Nerve-Center Jena, Jena University Hospital—Friedrich Schiller University, Am Klinikum 1, 07747 Jena, Germany; 4 Center for Rare Diseases, Jena University Hospital—Friedrich Schiller University, Am Klinikum 1, 07747 Jena, Germany

**Keywords:** artificial intelligence, deep learning, facial paralysis, image processing, computer-assisted

## Abstract

Peripheral facial palsy (PFP) causes pronounced facial asymmetry and functional impairment, highlighting the need for reliable, objective assessment. This study presents a novel, fully automated, reference-free method for quantifying facial symmetry using artificial intelligence (AI)-based facial landmark detection. A total of 405 datasets from 198 PFP patients were analyzed, each including nine standardized facial expressions covering both resting and dynamic movements. AI detected 478 landmarks per image, from which 225 paired landmarks were used to compute local asymmetry angles. Systematic evaluation identified 91 highly informative landmark pairs, primarily around the eyes, nose and mouth, which simplified the analysis and enhanced discriminatory power, while also enabling region-specific assessment of asymmetry. Statistical evaluation included Kruskal–Wallis H-tests across clinical scores and Spearman correlations, showing moderate to strong associations (0.32–0.73, *p* < 0.001). The fully automated pipeline produced reproducible results and demonstrated robustness to head rotation. Intuitive full-face angle maps allowed direct assessment of asymmetry without a reference image. This AI-driven approach provides a robust, objective, and visually interpretable framework for clinical monitoring, severity classification, and treatment evaluation in PFP, combining quantitative precision with practical applicability.

## 1. Introduction

Peripheral facial palsy (PFP) frequently results in pronounced facial asymmetry, functional limitations, and psychosocial impact [1,2,3,4,5,6,7]. Objective and reproducible assessment of facial symmetry is essential for monitoring recovery, guiding rehabilitation, and supporting clinical decision-making [8,9,10,11,12,13,14,15].

Artificial intelligence (AI)-based landmark approaches have become increasingly common for quantifying facial asymmetry, providing automated and reproducible measurements of facial features. For example, previous studies [16,17,18,19] automatically detected 68 facial landmarks to compute distance- or angle-based asymmetry indices, demonstrating good correlation with established clinical grading systems. Standardized head positioning and expression protocols have typically been required to ensure measurement reliability, which can limit applicability in clinical settings. A recent component-based model using dense facial landmarks correlates well with clinical scores after vestibular schwannoma surgery [20]. However, its visualizations may be unintuitive for clinicians or patients, potentially limiting practical interpretability. Previous work [21] introduced reference-based dynamic heatmaps as a robust approach for assessing facial asymmetry, using a neutral facial image as a reference and quantifying deviations in expressive facial states. This method provided reliable spatial mapping and objective scoring of asymmetrical regions, while being relatively insensitive to the precise localization of individual landmarks. By focusing on intra-individual changes relative to a personal neutral baseline, it captured dynamic facial movements and offered reproducible quantification suitable for clinical monitoring and research applications. However, despite these advances, a key limitation of existing approaches is that they may rely on a reference image, are not fully automated, or do not provide an intuitive representation of facial symmetry applicable across both resting and dynamic facial states. This limits their applicability in routine clinical settings and in scenarios where baseline images are unavailable.

Therefore, the aim of this study is to address this gap by developing and evaluating a fully automated, reference-free, landmark-based method for objective facial symmetry assessment in patients with PFP. By leveraging high-density facial landmarks and angle-based metrics, the proposed approach seeks to combine quantitative robustness with intuitive visualization, thereby extending current methods toward more flexible and clinically applicable use.

## 2. Materials and Methods

This retrospective study received approval from the Institutional Review Board of Jena University Hospital, Germany (protocol 2019-1539-BO). Written informed consent was not required due to the study design. All procedures adhered to the applicable ethical guidelines and the principles outlined in the Declaration of Helsinki.

### 2.1. Study Population and Image Acquisition

A total of 518 datasets from patients with unilateral PFP were retrospectively screened. They were collected between 26 March 2008 and 20 December 2011, and each consisted of nine standardized facial photographs capturing a range of expressions. Datasets were excluded if any of these nine images were missing. After applying these inclusion criteria, 405 datasets from 198 patients (94 female, 104 male) remained for analysis. Patient ages ranged from 4 to 90 years (mean 53 ± 19 years). Among these, 98 patients had multiple datasets acquired on different days during therapy.

Nine standardized facial photographs per patient captured a range of expressions, including:

(1) Neutral;
(2) Gentle eye closure;
(3) Forced eye closure;
(4) Frowning/forehead wrinkling;
(5) Nose wrinkling;
(6) Closed-mouth stretch;
(7) Mouth stretch with teeth visible;
(8) Lip pursing;
(9) Downward movement of the mouth corners.

Most images were taken with Nikon DSLR cameras (mainly D90 and D100), with a few captured using Canon or Sony cameras. Flash and white balance settings varied across cases, and image resolutions ranged from approximately 700 × 1000 to 1800 × 2400 pixels. Randomization and blinding were not applicable due to the retrospective, observational nature of the study.

### 2.2. Image Preprocessing and Analysis

**Figure 1:**
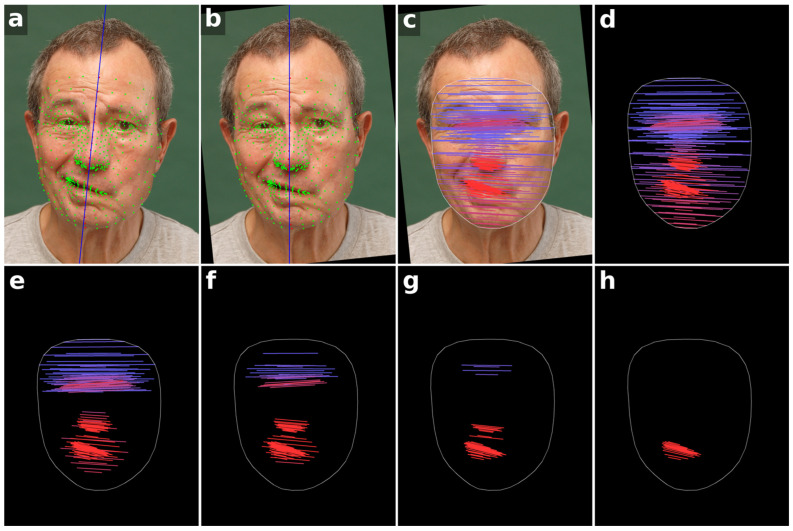
Workflow of the facial symmetry analysis method. (**a**) All 478 facial landmarks are initially detected (green and red points). A midline is estimated from the central red landmarks (blue line). (**b**) The image is rotated so that this midline becomes vertical, aligning the head horizontally. (**c**) Landmark pairs on the left and right sides of the face relative to the vertical midline are connected by lines, with color indicating the angle relative to the horizontal, transitioning from blue to red as the angle increases. (**d**) Visualization of the angle map without the patient photo, showing 225 landmark pairs. (**e**–**h**) Angle maps with progressively reduced landmark sets of 140, 91, 50, and 21 pairs, highlighting that the most informative features are concentrated in the mouth, nose and eye regions. Written informed consent for publication of these identifiable photographs was obtained from the patient.

Facial images were analyzed in Python (v3.14.0, Python Software Foundation) [22] using an AI-based landmark detection approach (MediaPipe v0.10.33, Google Research) [23], which automatically located 478 facial landmarks per photograph with enhanced prediction accuracy. To standardize head alignment, 20 central landmarks (excluding eight mouth landmarks) were first used to define a midline through the center of the face (see Figure 1a, red points). A straight line was fitted to these points using ordinary least squares regression to estimate the facial midline. Each image was then rotated around its center to achieve approximate horizontal head alignment (see Figure 1b).

For the analysis of facial symmetry, 225 landmark pairs were defined on corresponding left and right face locations (450 points total) to calculate the angles of the connecting lines relative to a horizontal reference (see Figure 1c). For each pair $L=\left(x_{L},y_{L}\right)$ and $R=\left(x_{R},y_{R}\right)$, the angle θ was calculated as:

(1) $$\theta =\;\frac{180}{\pi }\cdot arctan2\left(y_{R}-y_{L},x_{R}-x_{L}\right)$$

The set of all angles for a given image forms a high-dimensional asymmetry feature vector $a=(\theta_{1},\theta_{2},\ldots ,\theta_{N})$, representing local deviations for each landmark pair. Each angle encodes both direction and magnitude, providing a quantitative representation of facial asymmetry. Visual representations of these vectors were generated as angle maps (see Figure 1d).

From this asymmetry feature vector, a global asymmetry score was computed as the mean of the absolute angular deviations across all selected landmark pairs:

(2) $${Score}_{AI}=\;\frac{1}{N}\cdot \sum_{i=1}^{N}\left|\theta_{i}\right|$$

Higher Score_AI_ values indicate greater asymmetry. The reported standard deviations reflect variability of Score_AI_ across samples within each severity group. Region-wise features were also computed for the eyes, nose and mouth by selecting subsets of the vector, enabling localized assessment of asymmetry patterns.

The fully automated pipeline, including landmark detection, image rotation, angle computation, and visualization, produced reproducible results without manual correction or additional preprocessing (e.g., cropping, resizing, or intensity normalization).

To assess robustness, each image was rotated from −25° to 25° in 1° increments, and the previously described pipeline was applied to each rotation. The mean absolute deviation of Score_AI_ from the original image was then computed.

### 2.3. Landmark Filtering

In order to evaluate whether using fewer than 225 landmark pairs could improve the analysis (see Figure 1e–h), datasets with available Stennert index facial grading scores were examined. Of the 405 datasets, 266 included these scores and were used for landmark filtering and correlation analyses, while the remaining datasets were included only in the image-based symmetry analysis.

Facial nerve function was graded using the Stennert index, a commonly applied scoring system in Germany [24]. It evaluates facial symmetry both at rest (0 = no asymmetry, 4 = severe asymmetry) and during voluntary movement (0 = normal movement, 6 = complete or near-complete impairment).

For scoring at rest, four yes-or-no criteria are rated: Eyelid fissure difference 3 mm or more? Ectropion? Nasolabial fold smoothed (if formed on the healthy side)? Low corner of the mouth 3 mm or more? Every “yes” is counted with 1 point; every “no” with 0 points.

During movement, scoring is based on the following criteria: Frowning (wrinkle formation or raising of the eyebrow) not possible or only partly but less than 50% of the healthy side possible? Residual palpebral fissure in sleeping position? Residual palpebral fissure at tight eye closing? Teeth showing upper and lower canine teeth not visible? Teeth showing upper second incisor across entire width not visible? Corners of mouth (shortening of distance between philtrum and corner of mouth compared to healthy side) less than 50% compared to healthy side? Again, every “yes” is counted with 1 point; every “no” with 0 points.

One-way ANOVA was used to evaluate the discriminatory power of individual landmark pairs across Stennert index grades under resting and movement conditions. For each pair, absolute angular deviations were averaged across all images per patient to obtain patient-level values, and eta-squared (η^2^) was calculated to quantify the proportion of variance explained by grade as a measure of effect size. Systematic η^2^ thresholds (0.001–0.400 in 0.001 increments) were applied to identify the most informative pairs; only pairs exceeding the threshold in both conditions were retained. This threshold-based selection served as an exploratory feature-selection step.

To define the optimal number of landmark pairs, each subset of selected pairs was used to compute Score_AI_, which was then evaluated using the Kruskal–Wallis H-test across Stennert index grades to determine overall discriminatory power under both conditions. Finally, Spearman’s rank correlation coefficients were calculated between Score_AI_ derived from the optimal subset of landmark pairs and the corresponding clinical Stennert scores for resting and movement conditions, to assess the monotonic association between objective symmetry measures and clinical severity. Statistical significance was assessed using the corresponding *p*-values, with significance set at *p* < 0.05. All analyses were performed in Python using standard statistical libraries.

### 2.4. Comparison with Other 68-Point Landmark Models

For comparison with other landmark-based approaches, the standard Dlib 68-point predictor (shape_predictor_68_face_landmarks.dat) and three Emotrics-based 68-point predictors (mee_shape_predictor_68_face_landmarks.dat, mee_shape_predictor_68_face_landmarks2.dat, and mee_shape_predictor_68_face_landmarks3.dat) were evaluated. From each set, 6 landmarks excluding four mouth landmarks were used to define the facial midline for head alignment. For asymmetry analysis, 29 landmark pairs were initially defined and evaluated. In addition, a reduced set excluding landmarks corresponding to the facial contour was tested, resulting in 21 landmark pairs. All landmarks were processed using the same analytical pipeline as the 478-point model, including midline-based head alignment, left-right pairing, and Score_AI_ computation. This allowed a direct comparison between the 68-point configurations and the high-density 478-point model, which was also tested in reduced configurations of 140, 91, 50, and 21 landmark pairs.

## 3. Results

The method was successfully applied to all 405 datasets. Analyses requiring clinical Stennert index scores used a subset of 266 datasets. This subset allowed evaluation of the discriminatory power of landmark pair reductions in relation to clinical severity.

### 3.1. Discriminatory Power of Landmark Sets

**Figure 2:**
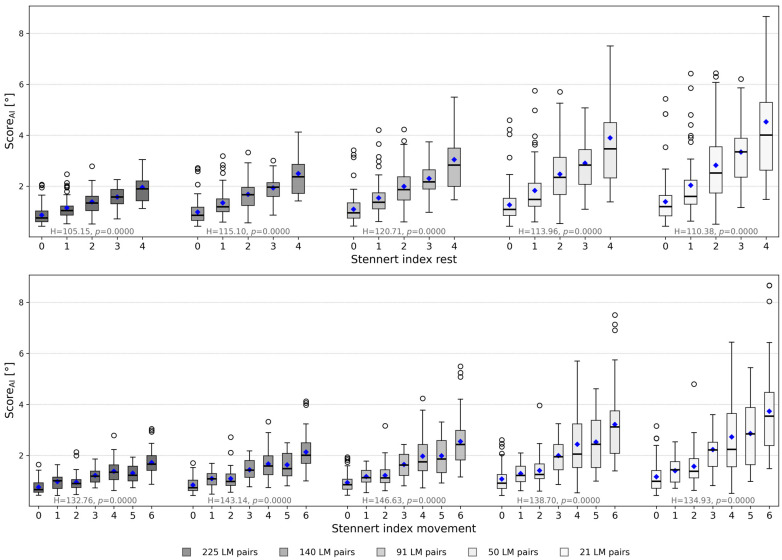
Discriminatory power of landmark sets across Stennert index grades. Boxplots show the distribution of Score_AI_ for each patient grouped by Stennert index levels at rest (**top**) and during voluntary movement (**bottom**) for five different landmark sets (225, 140, 91, 50, 21 pairs). The mean is marked with a blue diamond. The Kruskal–Wallis H-test statistic and associated *p*-value for each landmark set are indicated above the respective groups, reflecting the ability of each configuration to differentiate clinical severity. Larger H-values correspond to stronger separation between Stennert index grades, highlighting that the 91-pair configuration achieves optimal discriminatory performance.

To assess whether a reduced set of landmark pairs could differentiate between clinical severity levels, systematic reduction in landmark pairs was performed. The analysis showed that 91 pairs provided optimal separation between Stennert index grades. These pairs exhibited a minimum eta-squared (η^2^) value of 0.178, indicating a moderate to high discriminatory power across clinical severity levels. The Kruskal–Wallis H-test statistic initially increased with decreasing number of pairs, peaking at 91, and then declined as fewer pairs were included (Figure 2). Higher H-values indicate stronger separation of clinical severity levels, and *p*-values remained < 0.001 for all configurations, confirming statistically significant differentiation. These results suggest that the 91-pair configuration balances complexity and discriminatory performance effectively.

### 3.2. Score_AI_ Distribution and Correlation with Clinical Severity

**Figure 3:**
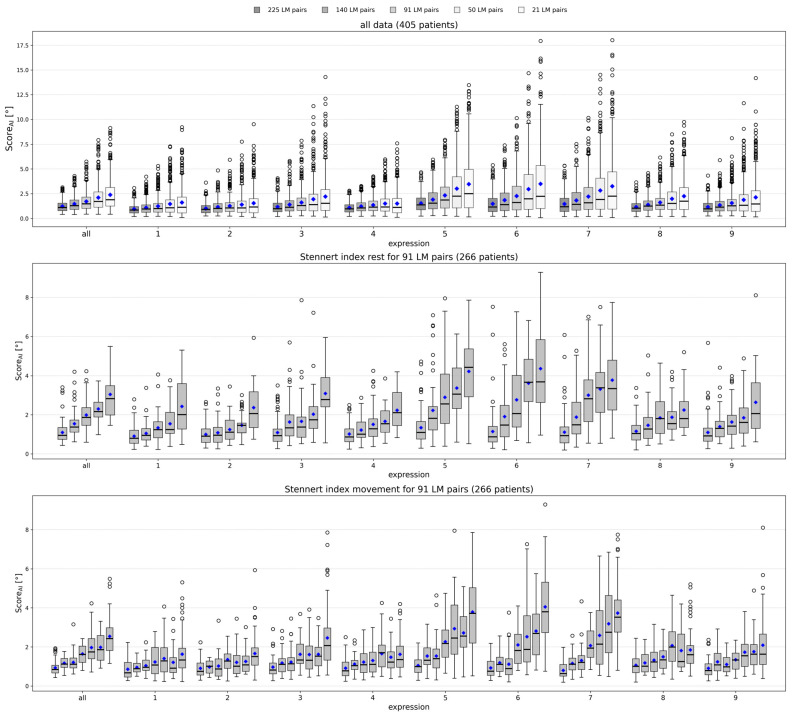
Distribution of Score_AI_ across facial expressions and Stennert index levels. Top: Boxplots of Score_AI_ for all nine expressions and the overall mean (“all”) for five landmark sets (225, 140, 91, 50, 21 pairs) across all patients, illustrating overall variability, with each set indicated by a different shade of gray. The mean is marked with a blue diamond. Middle: Boxplots for the 91-pair configuration, stratified by Stennert index at rest (levels 0–4), showing the distribution of Score_AI_ across the nine expressions and overall mean for patients with available clinical scores. Bottom: Boxplots for the 91-pair configuration, stratified by Stennert index during voluntary movement (levels 0–6), displaying the same information. Facial expressions: (1) neutral, (2) gentle eye closure, (3) forced eye closure, (4) frowning/forehead wrinkling, (5) nose wrinkling, (6) closed-mouth stretch, (7) mouth stretch with teeth visible, (8) lip pursing, and (9) downward movement of the mouth corners.

The relationship between Score_AI_ and clinical severity was examined across nine standardized facial expressions. For the 91 selected pairs, Score_AI_ increased with higher Stennert index grades, particularly for eye-, nose- and mouth-related movements. Expressions 5–7 under rest and movement showed the strongest correlation with clinical severity (Figure 3). This indicates that the method captures both static and dynamic asymmetry patterns. Summary statistics for all datasets (Table 1) and for the subset with Stennert scores (Table 2) further highlight that increasing clinical severity is associated with higher Score_AI_, reflecting reduced facial symmetry. Region-wise analyses for eye, nose and mouth landmarks are presented in Table A1, Table A2 and Table A3, respectively. These region-specific asymmetry scores allow evaluation of how each facial region contributes to overall facial asymmetry and correlates with clinical severity.

Spearman’s rank correlations confirmed moderate to strong associations between Score_AI_ derived from the 91 most informative landmark pairs and Stennert index scores (see Table 3). In addition, region-specific analyses based on the eye (24 pairs), nose (23 pairs), and mouth (44 pairs) subsets showed that the mouth region was more strongly correlated with clinical severity, particularly during voluntary movement, whereas eye-region correlations were lower but remained significant. The nose region also showed moderate to strong correlations for expressions 5–7; expression 4 (frowning/forehead wrinkling) showed a weak correlation at rest (r = 0.15, *p* = 0.013) and was not significant during movement (r = 0.06, *p* = 0.359). Mouth-related expressions (6–7) demonstrated the highest correlations during movement. Overall, these results indicate that the angular measures reflect facial asymmetry and that full-face and region-specific analyses provide complementary information.

### 3.3. Visualization

**Figure 4:**
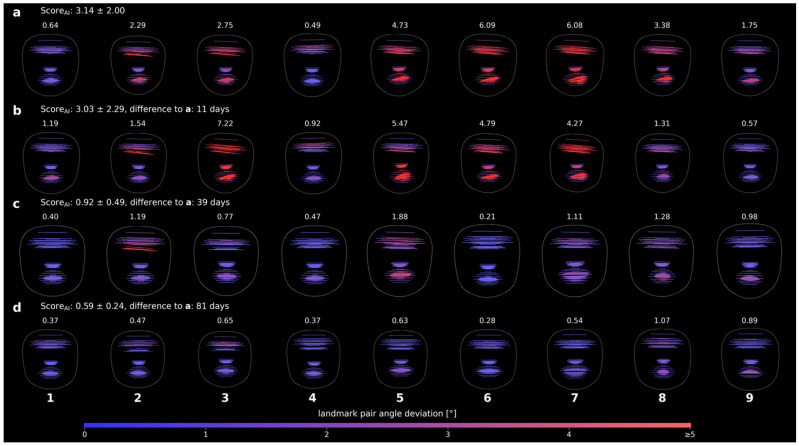
Visualization of facial symmetry in a single patient across multiple time points and nine standardized facial expressions. Each row corresponds to one time point, ordered chronologically (**a**–**d**), with the Score_AI_ of all 91 landmark pairs displayed above each row. Each column represents one facial expression (1–9), and the associated angle map is color-coded from blue (0° deviation) to red (≥5° deviation) to indicate the degree of asymmetry between paired landmarks. Facial expressions: (1) neutral, (2) gentle eye closure, (3) forced eye closure, (4) frowning/forehead wrinkling, (5) nose wrinkling, (6) closed-mouth stretch, (7) mouth stretch with teeth visible, (8) lip pursing, and (9) downward movement of the mouth corners.

**Figure 5:**
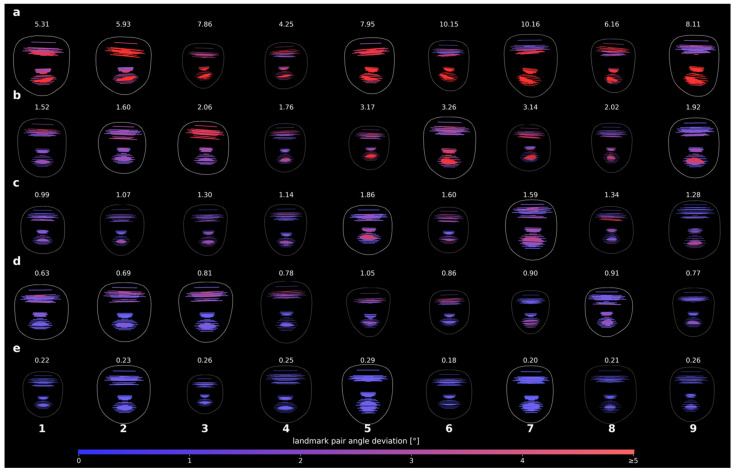
Visualization of facial symmetry across patients with peripheral facial palsy, showing extreme and percentile cases for each of the nine standardized facial expressions. Each row (**a**–**e**) represents one target group based on Score_AI_ values across 91 landmark pairs: maximum, 75th percentile, median, 25th percentile, and minimum. Columns correspond to the nine facial expressions (1–9). The angle maps are color-coded from blue (0° deviation) to red (≥5° deviation), indicating the degree of asymmetry between paired landmarks. Facial expressions: (1) neutral, (2) gentle eye closure, (3) forced eye closure, (4) frowning/forehead wrinkling, (5) nose wrinkling, (6) closed-mouth stretch, (7) mouth stretch with teeth visible, (8) lip pursing, and (9) downward movement of the mouth corners.

To examine whether changes over time and across patients could be visualized, angle maps were generated for both longitudinal and cross-sectional data. Example data from a single patient demonstrated that Score_AI_ and angle maps can track therapy-related improvements over time (Figure 4). Across the patient cohort, extreme and percentile cases illustrated the range of observed asymmetry patterns without revealing individual photographs (Figure 5). Dynamic expressions, such as mouth stretches, clearly highlighted affected sides, providing an intuitive visual representation of asymmetry across expression types.

### 3.4. Comparison with Other 68-Point Landmark-Based Models

**Figure 6:**
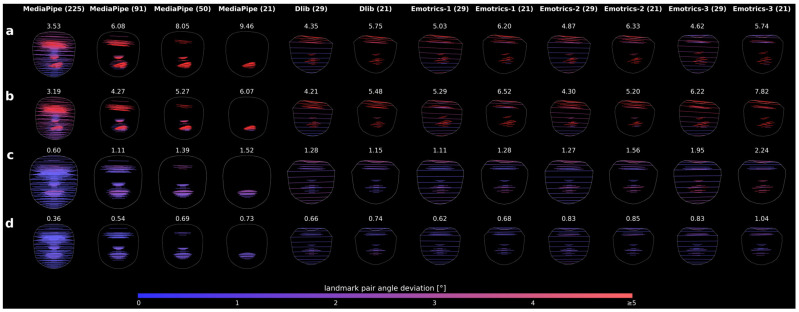
Comparison of landmark detection AI models using the same original image as in Figure 4, row 7. Rows (**a**–**d**) show different time points, and columns show the respective model configurations. The Score_AI_ is displayed above each image, and the color bar shows the angular deviation scale from 0° to ≥5°.

The reduced MediaPipe configurations demonstrated the strongest and most consistent separation across Stennert grades in both rest and movement. The 21- and 50-pair models achieved the highest slopes, indicating the greatest sensitivity to differences in severity, while the 91-pair configuration provided a favorable balance between slope and stability, maintaining informative regional patterns in a clinically interpretable format (see Figure 6). The full 225-pair MediaPipe model offered a more detailed representation but showed lower slopes, reflecting weaker separation between severity levels.

In comparison, Dlib- and Emotrics-based configurations generally exhibited lower slopes and greater variability in Score_AI_ across Stennert grades. Although some variants occasionally reached high maximum values, they showed less consistent increases across grades and wider ranges within individual severity levels, indicating less reliable differentiation (see Table 4 and Table A4). Across all methods, Score_AI_ increased with higher Stennert grades, confirming that the angular measures capture the progression of facial asymmetry.

### 3.5. Robustness to Artificial Rotations

The 91-pair Score_AI_ showed high stability across artificial head rotations from −25° to +25°. Absolute deviations of the Score_AI_ from the original (unrotated) images were low, with average deviations of 0.22 ± 0.21° and median deviations of 0.17° across the evaluated range. Even at the extremes of the rotation range, the Score_AI_ remained consistent, confirming robustness of the measure (see Table 5).

## 4. Discussion

The presented method provides a fully automated, reference-free approach for quantifying facial symmetry in patients with PFP, applicable to both resting and dynamic facial states. Angle-based measurements of paired AI-based landmarks provide a quantitative and reproducible measure, while visual angle maps allow intuitive identification of asymmetrical facial regions. Landmark pairs around the mouth, nose and eyes exerted the greatest influence on discriminating clinical Stennert index grades, which may reflect regions commonly affected in PFP, although this pattern may also be influenced by landmark distribution or expression-specific variability.

Automated assessment of facial palsy increasingly relies on landmark-based approaches. Lee et al. [16] used 68 CNN-detected landmarks to compute asymmetry scores from 10 to 12 distance ratios across six expressions in 128 patients, but were limited by a small, non-diverse training dataset (300 faces) and reliance on distance ratios. Kim et al. [17], Anping et al. [18], and Müller et al. [19] similarly applied 68 landmarks within Emotrics or Dlib frameworks to calculate linear and angular asymmetry parameters, yet all faced small cohorts (11–36 patients), limited expressions, and partial manual landmark adjustment. The present method leverages MediaPipe’s dense coverage of 478 facial points, trained on 30,000 in-the-wild images [25], enabling detailed spatial analysis and robust feature detection. This high-density mesh provides inherent redundancy as a key advantage for clinical robustness because localized errors in landmark detection or partial facial occlusions caused by hair or shadows are mitigated by averaging across the entire feature vector, which prevents single-point inaccuracies from disproportionately skewing the global asymmetry score. From 225 initial paired points, 91—mainly around the eyes, nose and mouth—were most discriminative for clinical grading. The Stennert index assesses facial regions in a way comparable to the Sunnybrook Facial Grading System, which has demonstrated high intra- and interrater reliability [14,26,27]. Although sensitivity and specificity are standard diagnostic metrics, they are not directly applicable here because the Stennert index is an ordinal rather than binary scale. Therefore, Spearman’s rank correlation was used to assess the association between angular measures and clinical severity. Effect size analysis confirmed that landmarks controlling eyelid closure, mouth opening, and lip corner movement carried the strongest discriminatory power, while regions such as the jaw contour or hairline contributed little, reflecting their limited functional relevance in facial palsy. The measured angles between these landmark pairs provide a quantitative assessment of facial asymmetry and serve as a proxy for surface-level manifestations of underlying muscle activation. While these geometric measures correlate with functional impairment, they reflect the cumulative effect of muscle tone and skin displacement rather than direct physiological activity. Guan et al. [20] reported similar findings with a component-based CNN for eyelid and mouth landmarks in 274 post-vestibular schwannoma patients, but their approach was restricted to discrete regions and did not allow an immediate, intuitive full-face visualization of asymmetry.

Compared to previous reference-based work [21], the current approach analyzes each image directly without requiring a neutral reference. In that earlier framework, expressive images were aligned to a neutral reference, and local spatial filtering was applied to the resulting difference maps to suppress minor landmark noise and emphasize dynamic deviations from the patient’s own baseline. This design proved highly robust when a neutral image was available. The new reference-free method, by contrast, computes angular symmetry measures directly from each frame, eliminating dependency on baseline alignment and potentially enabling application to single-image assessments, video sequences, retrospective photo collections, and telemedical use. These strategies address complementary aspects of the same problem: the reference-based pipeline enhances consistency and noise resilience, while the reference-free, high-density landmark approach improves spatial resolution and flexibility. The reference-based heatmap and the angle map each provide privacy-preserving, visually interpretable, and intuitive full-face visualizations of facial function.

The current framework is compatible with different AI-based landmark detection models, which may vary in performance depending on the application and over time as models are updated. Its dense landmark representation allows for both full-face and region-specific analyses by selecting corresponding subsets of the asymmetry feature vector, enabling localized assessment of asymmetry patterns without repeated image processing. In this study, full-face measurements integrating eye, nose, and mouth landmarks showed the highest overall discrimination across Stennert grades, whereas smaller configurations, such as the 21- and 50-pair MediaPipe sets, may perform particularly well when asymmetry is concentrated in specific regions, such as the mouth during voluntary movement. Because Score_AI_ is calculated as the mean of the absolute angular deviations across the selected landmark pairs, reduced subsets can emphasize region-specific effects, while broader configurations capture complementary asymmetry information across the face. A further advantage is that all analyses—including global scores, region-specific scores, and landmark-pair visualizations—can be derived simultaneously from a single image processing step, producing a comprehensive dataset for flexible and detailed interpretation of facial asymmetry. The framework also allows for future refinement of Score_AI_, such as weighting landmark pairs differently or integrating additional facial features, which could further enhance its discriminative power and clinical applicability.

More broadly, objective facial-performance measures should be interpreted in the context of underlying peripheral nerve function, muscle activation patterns, and rehabilitation status. Future studies could integrate image-based asymmetry metrics with complementary physiological measures, such as electromyography or clinical grading, and explore how neurobiological, cognitive, and stress-related factors influence facial motor patterns [28,29,30,31,32]. Such multimodal approaches may provide a more comprehensive interpretation of facial asymmetry beyond purely geometric analysis.

Despite its strengths, several limitations should be acknowledged. The accuracy of symmetry quantification depends directly on precise landmark localization and correct head orientation. To standardize image orientation, a vertical midline was defined using central facial landmarks, which is crucial because even small deviations from a horizontal head position can substantially distort angular measurements. Consequently, the accuracy of this alignment remains a critical factor influencing the overall precision of the symmetry metrics. Although the MediaPipe model, not specifically trained on PFP patients, generally performed robustly [21,33], extreme asymmetries or atypical expressions may still induce local errors that propagate to the angular measures. While robustness to moderate head rotation was confirmed through systematic sensitivity analysis, future studies could further quantify the impact of extreme facial occlusion, atypical expressions, or model-specific landmark noise, which can propagate to angular measurements. However, the current high-density framework is designed to minimize such effects through feature averaging. In practice, occlusion is avoided in patient recordings or images are retaken, and landmark variability depends on the AI model used. Additional variability arises from the retrospective dataset, including differences in camera type, resolution, and illumination. While midline alignment mitigates some of these effects, residual variations may still influence measurements. Moreover, as the method inherently compares the affected with the unaffected side, it cannot be directly applied to cases of bilateral facial palsy. Future studies could improve landmark detection by retraining the model with a large number of standardized PFP images or by implementing a secondary correction network to identify and adjust inconsistent landmarks. Achieving this, however, requires a substantial dataset of well-standardized images of PFP patients.

## 5. Conclusions

In conclusion, the proposed approach provides a fully automated and reference-free method for quantifying facial asymmetry in patients with PFP. Based on a high-density landmark model and a reduced set of 91 informative landmark pairs, which integrate eye, nose, and mouth landmarks, the method demonstrated moderate to strong associations with clinical Stennert index scores and effective discrimination between severity levels, highlighting the added value of combining complementary facial regions for assessing asymmetry. The resulting angle maps enable intuitive visualization of asymmetry patterns across different facial expressions, supporting the interpretation of regional differences. While the approach shows potential as an objective and reproducible framework for facial symmetry assessment, further studies are required to validate its clinical applicability and to compare its performance with established methods.

## Figures and Tables

**Table 1:** Summary statistics of Score_AI_ derived from 91 landmark pairs for each of the nine facial expressions and overall average (“all”) across all 405 datasets, as well as region-specific results for the eye, nose and mouth.

	Score_AI_ of Expressions [°]									
	1	2	3	4	5	6	7	8	9	All
mean	1.24	1.26	1.61	1.36	2.33	2.27	2.21	1.61	1.55	1.72
SD	0.86	0.81	1.18	0.79	1.67	1.88	1.77	0.99	1.11	0.96
variance	0.74	0.66	1.39	0.63	2.78	3.53	3.15	0.99	1.24	0.92
median	0.99	1.07	1.30	1.14	1.86	1.60	1.59	1.34	1.28	1.48
max	5.31	5.93	7.86	4.25	7.95	10.15	10.16	6.16	8.11	5.75
min	0.22	0.23	0.26	0.25	0.29	0.18	0.20	0.21	0.26	0.44
region-specific eye analysis (24 landmark pairs)										
mean	1.23	1.47	1.58	1.82	1.56	1.49	1.55	1.46	1.35	1.50
SD	0.77	1.0	1.08	1.2	1.07	1.09	1.11	0.97	0.88	0.70
region-specific nose analysis (23 landmark pairs)										
mean	0.83	0.81	1.01	0.85	1.77	1.54	1.69	1.06	1.07	1.18
SD	0.67	0.59	0.91	0.59	1.46	1.41	1.52	0.85	0.94	0.78
region-specific mouth analysis (44 landmark pairs)										
mean	1.45	1.38	1.94	1.38	3.04	3.07	2.85	1.99	1.92	2.12
SD	1.31	1.19	1.78	1.12	2.57	2.9	2.65	1.51	1.76	1.42

The table shows the mean, standard deviation (SD), variance, median, maximum (max), and minimum (min) symmetry scores. Facial expressions: (1) neutral, (2) gentle eye closure, (3) forced eye closure, (4) frowning/forehead wrinkling, (5) nose wrinkling, (6) closed-mouth stretch, (7) mouth stretch with teeth visible, (8) lip pursing, and (9) downward movement of the mouth corners.

**Table 2:** Score_AI_ (±standard deviation) derived from 91 landmark pairs, stratified by Stennert index for rest (0–4) and movement (0–6) across nine facial expressions. Higher Score_AI_ indicates lower facial symmetry between the affected and unaffected sides. A trend of increasing Score_AI_ with increasing Stennert index can be observed, particularly in nose wrinkling (expression 5) and mouth-related expressions (expressions 6 and 7) for movement, reflecting the association between higher facial palsy severity and reduced symmetry.

	n	Score_AI_ of Expressions [°]								
		1	2	3	4	5	6	7	8	9
Stennert index rest										
0	99	0.91 ± 0.51	1.00 ± 0.50	1.09 ± 0.66	1.01 ± 0.53	1.34 ± 0.86	1.14 ± 1.02	1.11 ± 0.88	1.16 ± 0.60	1.10 ± 0.74
1	53	1.05 ± 0.54	1.09 ± 0.66	1.63 ± 1.08	1.22 ± 0.61	2.23 ± 1.60	1.91 ± 1.32	1.88 ± 1.21	1.46 ± 0.92	1.40 ± 0.76
2	59	1.33 ± 0.72	1.25 ± 0.61	1.66 ± 1.18	1.51 ± 0.87	2.90 ± 1.66	2.77 ± 1.71	3.00 ± 1.74	1.84 ± 0.94	1.63 ± 0.90
3	39	1.54 ± 0.87	1.46 ± 0.62	2.03 ± 1.20	1.67 ± 0.69	3.37 ± 1.39	3.62 ± 1.47	3.31 ± 1.68	1.88 ± 0.90	1.85 ± 1.07
4	16	2.43 ± 1.49	2.37 ± 1.37	3.09 ± 1.59	2.23 ± 0.99	4.22 ± 2.02	4.37 ± 2.42	3.77 ± 2.05	2.25 ± 1.29	2.64 ± 1.99
Stennert index movement										
0	58	0.86 ± 0.48	0.91 ± 0.46	0.97 ± 0.57	0.91 ± 0.50	1.05 ± 0.47	0.92 ± 0.51	0.80 ± 0.43	1.06 ± 0.51	0.91 ± 0.46
1	27	0.96 ± 0.32	0.98 ± 0.34	1.17 ± 0.59	1.13 ± 0.48	1.54 ± 0.69	1.19 ± 0.55	1.15 ± 0.49	1.19 ± 0.53	1.24 ± 0.69
2	35	1.04 ± 0.42	1.02 ± 0.63	1.23 ± 0.70	1.23 ± 0.66	1.54 ± 0.95	1.12 ± 0.76	1.31 ± 0.74	1.32 ± 0.70	1.10 ± 0.45
3	21	1.23 ± 0.74	1.35 ± 0.64	1.62 ± 0.81	1.31 ± 0.66	2.27 ± 1.07	2.11 ± 1.07	2.07 ± 0.87	1.49 ± 0.73	1.35 ± 0.59
4	39	1.41 ± 0.86	1.21 ± 0.69	1.61 ± 0.93	1.66 ± 0.86	2.94 ± 1.66	2.53 ± 1.70	2.59 ± 1.74	2.07 ± 1.00	1.73 ± 0.95
5	22	1.21 ± 0.80	1.26 ± 0.65	1.63 ± 0.81	1.47 ± 0.79	2.72 ± 1.31	2.82 ± 1.41	3.18 ± 1.90	1.82 ± 1.10	1.76 ± 0.99
6	64	1.63 ± 1.09	1.66 ± 0.94	2.46 ± 1.60	1.62 ± 0.86	3.79 ± 1.82	4.05 ± 1.82	3.73 ± 1.58	1.85 ± 1.04	2.09 ± 1.44

The table also shows the number of datasets (n) used for each Stennert index category. Facial expressions: (1) neutral, (2) gentle eye closure, (3) forced eye closure, (4) frowning/forehead wrinkling, (5) nose wrinkling, (6) closed-mouth stretch, (7) mouth stretch with teeth visible, (8) lip pursing, and (9) downward movement of the mouth corners.

**Table 3:** Spearman’s rank correlation coefficients and corresponding *p*-values for the association between the Stennert index (rest and movement) and Score_AI_ derived from 91 landmark pairs across nine facial expressions in 266 datasets. Positive correlation coefficients indicate that higher clinical severity (higher Stennert index) is associated with higher Score_AI_, reflecting lower facial symmetry.

	Spearman’s Rank Correlation Coefficients of Expressions									
	1	2	3	4	5	6	7	8	9	All
rest	0.38	0.35	0.44	0.42	0.58	0.63	0.61	0.38	0.38	0.46
movement	0.32	0.38	0.49	0.36	0.64	0.71	0.73	0.36	0.44	0.49
*p*-value	<0.001									
region-specific eye analysis (24 landmark pairs)										
rest	0.24	0.33	0.41	0.45	0.22	0.35	0.39	0.28	0.24	0.32
movement	0.23	0.42	0.51	0.48	0.28	0.44	0.52	0.28	0.28	0.38
*p*-value	<0.001									
region-specific nose analysis (23 landmark pairs)										
rest	0.26	0.15	0.32	0.15	0.54	0.59	0.52	0.34	0.35	0.36
movement	0.18	0.13	0.31	0.06	0.59	0.61	0.63	0.35	0.39	0.36
*p*-value rest	<0.001	0.013	<0.001	0.013	<0.001					
*p*-value movement	0.003	0.040	<0.001	0.359						
region-specific mouth analysis (44 landmark pairs)										
rest	0.36	0.24	0.32	0.28	0.55	0.63	0.6	0.27	0.29	0.39
movement	0.3	0.23	0.34	0.22	0.61	0.71	0.71	0.26	0.34	0.41
*p*-value	<0.001									

Facial expressions: (1) neutral, (2) gentle eye closure, (3) forced eye closure, (4) frowning/forehead wrinkling, (5) nose wrinkling, (6) closed-mouth stretch, (7) mouth stretch with teeth visible, (8) lip pursing, and (9) downward movement of the mouth corners.

**Table 4:** Comparison of landmark-based methods for facial asymmetry across Stennert index grades. Score_AI_, along with range and slope, is shown for rest (Stennert 0–4) and movement (Stennert 0–6) conditions, with methods ranked by slope to indicate the strength of separation between severity levels.

At Rest (Stennert 0–4)				During Facial Movement (Stennert 0–6)			
Ranked Method (LM Pairs)	Score_AI_ [°]	Range	Slope	Ranked Method (LM Pairs)	Score_AI_ [°]	Range	Slope
MediaPipe (21)	2.82 ± 0.59	3.14	0.76	MediaPipe (21)	2.24 ± 0.49	2.58	0.42
MediaPipe (50)	2.47 ± 1.04	2.63	0.63	MediaPipe (50)	1.99 ± 0.86	2.14	0.35
Emotrics-2 (21)	2.95 ± 2.22	1.99	0.47	Emotrics-1 (21)	2.80 ± 2.13	1.72	0.29
MediaPipe (91)	1.99 ± 1.21	1.95	0.47	Emotrics-2 (21)	2.59 ± 1.93	1.68	0.26
Emotrics-1 (21)	3.10 ± 2.34	1.76	0.41	MediaPipe (91)	1.64 ± 1.00	1.61	0.26
Dlib (21)	2.42 ± 1.48	1.73	0.39	Emotrics-3 (21)	2.74 ± 2.14	1.40	0.25
Emotrics-2 (29)	2.49 ± 2.11	1.57	0.37	Emotrics-1 (29)	2.32 ± 2.03	1.34	0.23
MediaPipe (140)	1.69 ± 1.17	1.51	0.36	Dlib (21)	2.12 ± 1.29	1.50	0.22
Emotrics-3 (21)	3.01 ± 2.36	1.46	0.35	Emotrics-2 (29)	2.21 ± 1.83	1.32	0.21
Dlib (29)	2.13 ± 1.47	1.40	0.32	MediaPipe (140)	1.41 ± 0.96	1.29	0.20
Emotrics-1 (29)	2.56 ± 2.24	1.38	0.32	Emotrics-3 (29)	2.27 ± 2.03	1.07	0.19
Emotrics-3 (29)	2.48 ± 2.24	1.13	0.27	Dlib (29)	1.89 ± 1.28	1.20	0.18
MediaPipe (225)	1.39 ± 1.07	1.10	0.26	MediaPipe (225)	1.18 ± 0.88	0.97	0.14

**Table 5:** Absolute deviations of the Score_AI_ (derived from the 91-pair asymmetry feature vector) from the original images across selected artificial head rotations for all 405 datasets.

Image Rotation [°]	Deviation of Score_AI_ from Original Image [°]		Image Rotation [°]	Deviation of Score_AI_ from Original Image [°]	
	Mean	Median		Mean	Median
1	0.15 ± 0.14	0.11	−1	0.15 ± 0.14	0.11
5	0.18 ± 0.16	0.14	−5	0.18 ± 0.17	0.13
10	0.21 ± 0.19	0.16	−10	0.21 ± 0.18	0.16
15	0.24 ± 0.21	0.18	−15	0.23 ± 0.21	0.18
20	0.27 ± 0.24	0.20	−20	0.26 ± 0.23	0.19
25	0.29 ± 0.27	0.22	−25	0.29 ± 0.26	0.22

Mean and median deviations are shown for representative rotations.

## Data Availability

The datasets analyzed during the current study are not publicly available due to privacy concerns. However, all relevant data supporting the findings of this study are included in the article. For further inquiries or data requests, please contact the corresponding author.

## Abbreviations

The following abbreviations are used in this manuscript:

AI	Artificial Intelligence
CNN	Convolutional Neural Network
PFP	Peripheral facial palsy

## Appendix A

**Table A1:** Score_AI_ (±standard deviation) derived from 24 landmark pairs for the eye region, stratified by Stennert index for rest (0–4) and movement (0–6) across nine facial expressions.

	n	Score_AI_ (Eye Region) of Expressions [°]								
		1	2	3	4	5	6	7	8	9
Stennert index rest										
0	99	1.02 ± 0.64	1.13 ± 0.79	1.09 ± 0.81	1.07 ± 0.73	1.29 ± 0.96	1.11 ± 0.76	1.08 ± 0.86	1.13 ± 0.74	1.10 ± 0.71
1	53	1.31 ± 0.82	1.43 ± 0.93	1.63 ± 1.03	2.06 ± 1.20	1.55 ± 0.98	1.42 ± 0.89	1.48 ± 1.00	1.52 ± 0.94	1.23 ± 0.75
2	59	1.31 ± 0.77	1.59 ± 0.84	1.78 ± 1.00	2.07 ± 1.23	1.83 ± 1.22	1.56 ± 0.97	1.79 ± 1.16	1.69 ± 1.05	1.42 ± 0.88
3	39	1.28 ± 0.69	1.71 ± 0.92	2.10 ± 1.27	2.23 ± 1.00	1.82 ± 1.11	2.12 ± 1.57	2.23 ± 1.59	1.60 ± 0.91	1.48 ± 0.91
4	16	2.11 ± 1.01	2.82 ± 1.55	2.87 ± 1.49	2.34 ± 1.26	2.01 ± 1.30	2.73 ± 1.11	2.51 ± 0.82	2.19 ± 0.98	2.26 ± 1.28
Stennert index movement										
0	58	0.89 ± 0.60	0.90 ± 0.61	0.85 ± 0.50	0.87 ± 0.62	1.06 ± 0.75	0.92 ± 0.61	0.76 ± 0.54	0.99 ± 0.66	0.88 ± 0.63
1	27	1.31 ± 0.74	1.30 ± 0.83	1.35 ± 0.92	1.46 ± 0.68	1.59 ± 1.02	1.38 ± 0.84	1.37 ± 1.00	1.52 ± 0.85	1.43 ± 0.81
2	35	1.27 ± 0.88	1.21 ± 0.96	1.19 ± 0.81	1.66 ± 1.39	1.48 ± 0.91	1.13 ± 0.80	1.22 ± 0.79	1.37 ± 0.90	1.19 ± 0.72
3	21	1.25 ± 0.68	1.84 ± 0.81	1.66 ± 0.60	1.71 ± 1.20	1.44 ± 0.91	1.47 ± 0.93	1.47 ± 0.90	1.47 ± 1.20	1.22 ± 0.75
4	39	1.31 ± 0.75	1.41 ± 0.79	1.57 ± 0.95	2.37 ± 1.28	1.88 ± 1.21	1.51 ± 0.99	1.59 ± 1.12	1.82 ± 1.05	1.54 ± 0.94
5	22	1.33 ± 0.59	1.60 ± 0.98	2.02 ± 1.07	2.05 ± 0.92	1.38 ± 0.87	1.36 ± 0.73	1.74 ± 0.99	1.30 ± 0.76	1.25 ± 0.59
6	64	1.46 ± 0.90	2.11 ± 1.13	2.50 ± 1.34	2.19 ± 1.00	2.06 ± 1.29	2.40 ± 1.34	2.56 ± 1.33	1.76 ± 0.93	1.67 ± 1.06

The table also shows the number of datasets (n) used for each Stennert index category. Facial expressions: (1) neutral, (2) gentle eye closure, (3) forced eye closure, (4) frowning/forehead wrinkling, (5) nose wrinkling, (6) closed-mouth stretch, (7) mouth stretch with teeth visible, (8) lip pursing, and (9) downward movement of the mouth corners.

**Table A2:** Score_AI_ (±standard deviation) derived from 23 landmark pairs for the nose region, stratified by Stennert index for rest (0–4) and movement (0–6) across nine facial expressions.

	n	Score_AI_ (Nose Region) of Expressions [°]								
		1	2	3	4	5	6	7	8	9
Stennert index rest										
0	99	0.63 ± 0.42	0.70 ± 0.43	0.68 ± 0.50	0.77 ± 0.49	0.93 ± 0.64	0.78 ± 0.74	0.84 ± 0.73	0.74 ± 0.52	0.71 ± 0.59
1	53	0.68 ± 0.53	0.76 ± 0.63	1.01 ± 0.98	0.70 ± 0.46	1.72 ± 1.55	1.24 ± 1.03	1.49 ± 1.14	0.96 ± 0.89	0.91 ± 0.70
2	59	0.84 ± 0.57	0.74 ± 0.51	0.91 ± 0.79	0.87 ± 0.65	2.22 ± 1.22	1.87 ± 1.34	2.23 ± 1.51	1.27 ± 0.73	1.14 ± 0.81
3	39	1.03 ± 0.83	0.89 ± 0.54	1.32 ± 0.99	1.01 ± 0.71	2.63 ± 1.50	2.53 ± 1.11	2.45 ± 1.53	1.32 ± 0.89	1.36 ± 0.92
4	16	1.51 ± 1.12	1.27 ± 0.87	1.93 ± 1.23	1.44 ± 0.85	3.07 ± 1.94	2.78 ± 1.83	2.98 ± 1.97	1.49 ± 1.28	1.84 ± 1.57
Stennert index movement										
0	58	0.63 ± 0.43	0.69 ± 0.45	0.67 ± 0.56	0.73 ± 0.47	0.77 ± 0.49	0.71 ± 0.44	0.65 ± 0.39	0.69 ± 0.48	0.62 ± 0.37
1	27	0.62 ± 0.30	0.66 ± 0.29	0.65 ± 0.32	0.78 ± 0.50	1.01 ± 0.57	0.75 ± 0.46	0.79 ± 0.41	0.71 ± 0.33	0.71 ± 0.57
2	35	0.65 ± 0.35	0.75 ± 0.49	0.81 ± 0.58	0.80 ± 0.47	0.98 ± 0.65	0.69 ± 0.44	0.95 ± 0.65	0.72 ± 0.44	0.63 ± 0.41
3	21	0.78 ± 0.55	0.75 ± 0.55	0.98 ± 0.70	0.89 ± 0.70	1.79 ± 1.03	1.37 ± 0.90	1.50 ± 0.76	1.05 ± 0.74	0.97 ± 0.47
4	39	0.98 ± 0.70	0.79 ± 0.64	0.96 ± 0.78	1.04 ± 0.67	2.49 ± 1.27	1.82 ± 1.34	2.03 ± 1.60	1.44 ± 0.83	1.22 ± 0.87
5	22	0.80 ± 0.67	0.87 ± 0.48	0.92 ± 0.53	0.90 ± 0.61	2.15 ± 1.27	1.95 ± 1.11	2.59 ± 1.72	1.22 ± 0.88	1.15 ± 0.80
6	64	1.00 ± 0.89	0.91 ± 0.70	1.46 ± 1.28	0.89 ± 0.73	2.77 ± 1.76	2.64 ± 1.49	2.77 ± 1.47	1.33 ± 1.05	1.52 ± 1.20

The table also shows the number of datasets (n) used for each Stennert index category. Facial expressions: (1) neutral, (2) gentle eye closure, (3) forced eye closure, (4) frowning/forehead wrinkling, (5) nose wrinkling, (6) closed-mouth stretch, (7) mouth stretch with teeth visible, (8) lip pursing, and (9) downward movement of the mouth corners.

**Table A3:** Score_AI_ (±standard deviation) derived from 44 landmark pairs for the mouth region, stratified by Stennert index for rest (0–4) and movement (0–6) across nine facial expressions.

	n	Score_AI_ (Mouth Region) of Expressions [°]								
		1	2	3	4	5	6	7	8	9
Stennert index rest										
0	99	1.00 ± 0.73	1.07 ± 0.71	1.31 ± 1.03	1.11 ± 0.81	1.58 ± 1.23	1.35 ± 1.53	1.27 ± 1.23	1.39 ± 0.88	1.30 ± 1.14
1	53	1.11 ± 0.76	1.07 ± 0.90	1.95 ± 1.64	1.03 ± 0.84	2.87 ± 2.48	2.53 ± 2.04	2.30 ± 1.76	1.70 ± 1.36	1.74 ± 1.26
2	59	1.60 ± 1.14	1.32 ± 1.00	1.99 ± 1.90	1.54 ± 1.21	3.83 ± 2.64	3.89 ± 2.65	4.07 ± 2.65	2.23 ± 1.39	2.01 ± 1.44
3	39	1.96 ± 1.31	1.63 ± 1.07	2.36 ± 1.86	1.70 ± 1.13	4.60 ± 2.20	5.01 ± 2.22	4.36 ± 2.46	2.32 ± 1.65	2.30 ± 1.88
4	16	3.09 ± 2.24	2.70 ± 2.04	3.83 ± 2.47	2.58 ± 1.70	6.03 ± 3.26	6.09 ± 3.94	4.88 ± 3.22	2.67 ± 1.86	3.27 ± 3.18
Stennert index movement										
0	58	0.96 ± 0.69	1.02 ± 0.68	1.19 ± 0.96	1.03 ± 0.77	1.20 ± 0.77	1.04 ± 0.76	0.90 ± 0.70	1.30 ± 0.76	1.07 ± 0.73
1	27	0.94 ± 0.47	0.96 ± 0.57	1.35 ± 0.92	1.12 ± 0.81	1.78 ± 1.10	1.32 ± 0.72	1.23 ± 0.71	1.25 ± 0.78	1.41 ± 1.06
2	35	1.13 ± 0.66	1.06 ± 0.85	1.47 ± 1.06	1.21 ± 0.95	1.85 ± 1.43	1.33 ± 1.14	1.55 ± 1.11	1.61 ± 1.09	1.31 ± 0.74
3	21	1.46 ± 1.14	1.40 ± 0.94	1.94 ± 1.33	1.31 ± 0.94	2.97 ± 1.51	2.83 ± 1.58	2.69 ± 1.29	1.74 ± 0.99	1.62 ± 1.00
4	39	1.68 ± 1.29	1.32 ± 1.10	1.98 ± 1.44	1.60 ± 1.19	3.75 ± 2.63	3.45 ± 2.57	3.42 ± 2.53	2.53 ± 1.59	2.11 ± 1.57
5	22	1.36 ± 1.17	1.27 ± 1.09	1.78 ± 1.26	1.46 ± 1.09	3.75 ± 2.15	4.06 ± 2.29	4.28 ± 2.95	2.41 ± 1.71	2.35 ± 1.75
6	64	2.05 ± 1.61	1.82 ± 1.40	2.96 ± 2.52	1.70 ± 1.40	5.26 ± 2.89	5.68 ± 2.87	4.88 ± 2.49	2.17 ± 1.59	2.61 ± 2.31

The table also shows the number of datasets (n) used for each Stennert index category. Facial expressions: (1) neutral, (2) gentle eye closure, (3) forced eye closure, (4) frowning/forehead wrinkling, (5) nose wrinkling, (6) closed-mouth stretch, (7) mouth stretch with teeth visible, (8) lip pursing, and (9) downward movement of the mouth corners.

**Table A4:** Score_AI_ is reported for each landmark-based method, including the proposed MediaPipe configurations, as well as Dlib and Emotrics variants with 21 and 29 landmark (LM) pairs. Values are shown for rest (Stennert 0–4) and movement (Stennert 0–6) conditions.

Score_AI_ [°]												
	MediaPipe				Dlib		Emotrics-1		Emotrics-2		Emotrics-3	
LM	225	91	50	21	29	21	29	21	29	21	29	21
Stennert index rest												
0	0.86 ± 0.58	1.10 ± 0.67	1.27 ± 0.59	1.39 ± 0.34	1.43 ± 0.94	1.55 ± 0.93	1.82 ± 1.58	2.16 ± 1.66	1.68 ± 1.34	1.93 ± 1.41	1.86 ± 1.60	2.21 ± 1.69
1	1.15 ± 0.83	1.54 ± 0.95	1.83 ± 0.84	2.03 ± 0.47	1.92 ± 1.31	2.17 ± 1.32	2.39 ± 2.11	2.88 ± 2.22	2.24 ± 1.84	2.62 ± 1.93	2.35 ± 2.15	2.85 ± 2.27
2	1.38 ± 1.07	1.99 ± 1.20	2.47 ± 1.03	2.82 ± 0.59	2.18 ± 1.49	2.47 ± 1.49	2.58 ± 2.22	3.10 ± 2.32	2.50 ± 2.10	2.94 ± 2.21	2.44 ± 2.15	2.94 ± 2.27
3	1.58 ± 1.27	2.30 ± 1.44	2.90 ± 1.22	3.34 ± 0.73	2.29 ± 1.58	2.65 ± 1.58	2.81 ± 2.55	3.44 ± 2.66	2.79 ± 2.39	3.35 ± 2.50	2.77 ± 2.61	3.40 ± 2.75
4	1.96 ± 1.61	3.04 ± 1.79	3.89 ± 1.52	4.53 ± 0.84	2.82 ± 2.04	3.28 ± 2.07	3.21 ± 2.74	3.92 ± 2.84	3.25 ± 2.91	3.92 ± 3.05	2.99 ± 2.71	3.67 ± 2.84
Stennert index movement												
0	0.75 ± 0.49	0.93 ± 0.56	1.07 ± 0.49	1.16 ± 0.27	1.30 ± 0.85	1.38 ± 0.84	1.66 ± 1.40	1.93 ± 1.48	1.55 ± 1.23	1.75 ± 1.31	1.81 ± 1.53	2.13 ± 1.61
1	0.97 ± 0.62	1.17 ± 0.71	1.29 ± 0.63	1.40 ± 0.34	1.58 ± 1.04	1.73 ± 1.05	1.89 ± 1.63	2.27 ± 1.69	1.81 ± 1.46	2.10 ± 1.54	1.88 ± 1.61	2.25 ± 1.69
2	0.95 ± 0.67	1.21 ± 0.76	1.41 ± 0.68	1.57 ± 0.36	1.75 ± 1.19	1.99 ± 1.18	2.03 ± 1.80	2.44 ± 1.90	2.07 ± 1.69	2.44 ± 1.77	2.04 ± 1.83	2.46 ± 1.93
3	1.21 ± 0.85	1.64 ± 0.96	1.99 ± 0.83	2.23 ± 0.50	1.83 ± 1.20	2.03 ± 1.19	2.29 ± 1.99	2.75 ± 2.09	2.10 ± 1.65	2.46 ± 1.72	1.99 ± 1.73	2.39 ± 1.82
4	1.37 ± 1.04	1.97 ± 1.17	2.43 ± 1.00	2.72 ± 0.60	2.09 ± 1.42	2.40 ± 1.42	2.55 ± 2.25	3.10 ± 2.36	2.52 ± 2.14	2.99 ± 2.25	2.42 ± 2.22	2.94 ± 2.34
5	1.30 ± 1.11	1.98 ± 1.25	2.52 ± 1.08	2.86 ± 0.60	2.15 ± 1.55	2.41 ± 1.59	2.84 ± 2.48	3.43 ± 2.61	2.56 ± 2.18	2.99 ± 2.32	2.88 ± 2.71	3.53 ± 2.87
6	1.72 ± 1.39	2.54 ± 1.57	3.21 ± 1.34	3.73 ± 0.78	2.50 ± 1.73	2.88 ± 1.75	3.00 ± 2.65	3.65 ± 2.77	2.86 ± 2.46	3.43 ± 2.58	2.83 ± 2.59	3.46 ± 2.73
